# Disability Among School Children Across Districts of India

**DOI:** 10.1001/jamanetworkopen.2025.17223

**Published:** 2025-06-25

**Authors:** Janny Liao, Akhil Kumar, Rockli Kim, S. V. Subramanian

**Affiliations:** 1 Harvard College, Harvard University, Cambridge, Massachusetts; 2 Faculty of Arts and Sciences, University of Toronto, Toronto, Ontario, Canada; 3 Division of Health Policy and Management, College of Health Science, Korea University, Seoul, South Korea; 4 Interdisciplinary Program in Precision Public Health, Department of Public Health Sciences, Korea University, Seoul, South Korea; 5 Department of Social and Behavioral Sciences, Harvard T. H. Chan School of Public Health, Boston, Massachusetts; 6 Harvard Center for Population and Development Studies, Cambridge, Massachusetts

## Abstract

This cross-sectional study of children with special needs in India examines distributions of disability type by region and gender.

## Introduction

Disability is a significant challenge among children in India, affecting their quality of life, health outcomes, educational attainment, and socioeconomic status.^1^ Intellectual disabilities are particularly prevalent among children, and especially adolescents.^2^ However, disability is often underreported in India due to illiteracy, low awareness, cultural stigma, and limited access to health care and diagnostic services.^3^ There is a notable gap for robust estimates of population disability headcounts among school-aged children, typically provided through surveys only as an estimated prevalence. The last census that collected disability information from household members was done in 2011. Therefore, we utilized a novel dataset covering all schools in India that provides the headcount of children with special needs (CWSN) enrolled during the 2023-2024 school year.^4^ We analyzed the distribution of disabilities across categories and present these findings by gender and across 733 districts.^4,5^

## Methods

### Data Source

This study followed the Strengthening the Reporting of Observational Studies in Epidemiology (STROBE) reporting guideline. We used administrative education data from the Unified District Information System for Education Plus (UDISE+) released by the Government of India’s Department of School Education and Literacy within the Ministry of Education.^4^ The dataset, which consists of responses from a voluntary survey sent to all schools in India, is updated yearly from over 1.47 million schools. This survey is completed by designated *nodal officers*, which include principals, vice principals, head masters, head teachers, or the most senior teacher at each school.^4^ Due to district data availability, data for school year 2023-2024 was only used for the national summary and data from school year 2021-2022 was used for the district-level analysis. National analysis and district-level analysis included all children enrolled in any school with an active UDISE+ code as of March 2024 and September 2021, respectively. This study was exempted from institutional review board review and informed consent requirements, as determined by the Harvard Longwood Campus IRB Self-Determination Decision Tool, because it does not meet the definition of human participant research.

The outcomes of interest were the headcount of CWSN broadly classified into 5 categories following the definitions outlined in the 2016 Rights of Persons with Disabilities Act: physical disabilities, intellectual disabilities, mental disabilities, neurological and hematological disabilities, and multiple disabilities.^5^ We present the counts of children with disabilities and do not make any adjustments to the data extracted from the UDISE+ system. We assessed geographic variability by generating box plots that compared median values, IQRs, and extremes across regions for each impairment. Gender differences were assessed by comparing overall headcounts with disease-specific counts for boys and girls.

## Results

In total, there were 248 045 828 children enrolled in 1 471 891 schools in the 2023-2024 academic school year. A total of 2 114 110 children were registered as CWSN, reflecting an overall prevalence of 0.85% (Table). The gender distribution consisted of 1 210 672 boys and 903 438 girls, reflecting a total 14.6% higher burden for boys. Conditions with the greatest gender differences in reporting for boys were hemophilia (1606 boys [73.4%] vs 581 girls [26.6%]), autism spectrum disorder (17 199 boys [73.3%] vs 6250 girls [26.7%]), and muscular dystrophy (14 215 boys [63.6%] vs 8150 girls [36.4%]). In contrast, low vision showed the smallest gender difference at 1.0% (141 375 boys [49.5%] vs 144 484 girls [50.5%]) (Table).

**Table.** Headcount and Burden of Children With Special Needs by Impairment Type in India, 2024^a^

Impairment type	Children with special needs, No. (%)^b^		
	Overall (N = 2 114 110)	Boys (n = 1 210 672)	Girls (n = 903 438)
Intellectual disability	390 746 (18.5)	231 981 (59.4)	158 765 (40.6)
Locomotor disability	325 070 (15.4)	191 054 (58.8)	134 016 (41.2)
Low vision	285 859 (13.5)	141 375 (49.5)	144 484 (50.5)
Specific learning disabilities	271 642 (12.9)	155 132 (57.1)	116 510 (42.9)
Speech and language	215 463 (10.2)	128 653 (59.7)	86 810 (40.3)
Hearing impairment	208 860 (9.9)	114 946 (55.0)	93 914 (45.0)
Mental illness	111 242 (5.3)	66 021 (59.3)	45 221 (40.7)
Cerebral palsy	78 219 (3.7)	47 486 (60.7)	30 733 (39.3)
Multiple disability (including deafness and blindness)	75 939 (3.6)	45 445 (59.8)	30 494 (40.2)
Blindness	48 811 (2.3)	27 507 (56.3)	21 304 (43.7)
Autism spectrum disorder	23 449 (1.1)	17 199 (73.3)	6250 (26.7)
Muscular dystrophy	22 365 (1.1)	14 215 (63.6)	8150 (36.4)
Sickle cell disease	16 199 (0.8)	7752 (47.9)	8447 (52.1)
Dwarfism	13 607 (0.6)	6289 (46.2)	7318 (53.8)
Thalassemia	7851 (0.4)	4407 (56.1)	3444 (43.9)
Chronic neurological conditions	6436 (0.3)	3795 (59.0)	2641 (41.0)
Multiple sclerosis	6373 (0.3)	3753 (58.9)	2620 (41.1)
Hemophilia	2187 (0.1)	1606 (73.4)	581 (26.6)
Leprosy cured students	1846 (0.1)	975 (52.8)	871 (47.2)
Parkinson disease	1243 (0.1)	707 (56.9)	536 (43.1)
Acid attack victim	703 (<0.1)	374 (53.2)	329 (46.8)

^a^ Disabilities were classified into 5 categories for analysis: physical disabilities (including leprosy cured, cerebral palsy, dwarfism, muscular dystrophy, effects of acid attacks, blindness, hearing impairment, speech and language disabilities, locomotor disability, and low vision), intellectual disabilities (including specific learning disabilities and autism spectrum disorder), mental disabilities (including mental illness), neurological and hematological disabilities (including multiple sclerosis, Parkinson disease, hemophilia, thalassemia, sickle cell disease, and chronic neurological conditions), and multiple disabilities.

^b^ Overall burden indicates the percentage of disease-specific counts relative to the total count of children with special needs. Gender-specific burden indicates the percentage of girls and boys relative to the total count of children with each type of impairment.

For district-level analysis, we used 2021-2022 UDISE+ data, which included 2 243 480 children (after excluding 23 312 children with unavailable district information). There was significant variation between districts for intellectual and physical disabilities as demonstrated by wide IQRs (Figure). Specific learning disabilities had the highest median counts with wide IQRs, suggesting that it has the larger geographic variability across districts. In contrast, neurological and hematological related conditions had smaller differences.

**Figure.**
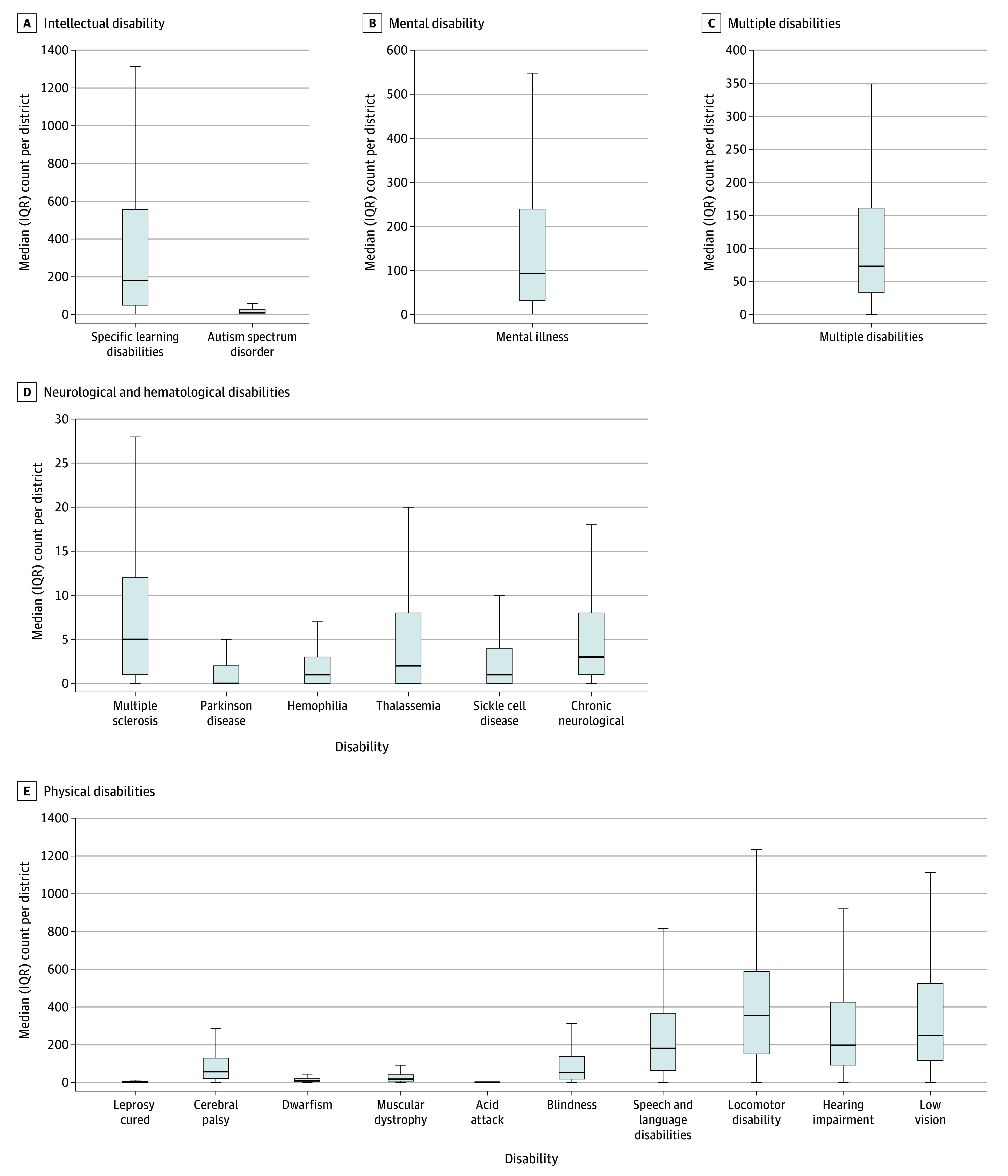
Summary of Impairment Type Distribution Across 733 Districts in India, 2022 We excluded 23 312 children with special needs due to unavailable district information. The horizontal bar inside the boxes indicates the median. The lower and upper ends of the boxes are the first and third quartiles. The whiskers indicate values within 1.5 times the IQR from the upper or lower quartile.

## Discussion

Our study highlights a significant burden of over 2.1 million school-aged CWSN. The overall burden was relatively evenly distributed across genders, except for autism spectrum disorder, hemophilia, and muscular dystrophy, which had a higher headcount reported among boys. This gender differences aligns with existing research, where boys generally exhibit higher reporting rates than girls. A key limitation of this study is that the data are based on voluntary counts reported by school officials who may not be clinically trained. Thus, classifications may vary across schools and limit the validity and consistency of reported disability types and headcounts. Understanding variations of disabilities geographically, by gender, and by type could guide resource allocation and policy development to better support CWSN in India.

## Notes

Supplement.
Data Sharing Statement
