# Error in Byline

**DOI:** 10.1001/jamanetworkopen.2025.28087

**Published:** 2025-07-22

In the Research Letter titled “Disability Among School Children Across Districts of India,”^1^ published June 25, 2025, there was an error in the degree listed for Janny Liao. This article has been corrected.^1^
