# Supplementary material for: Disability Among School Children Across Districts of India
Source: JAMA Netw Open. 2025 Jun 25;8(6):e2517223. doi: 10.1001/jamanetworkopen.2025.17223 (PMC12199054; doi:10.1001/jamanetworkopen.2025.17223)
Supplement: Supplement. — Data Sharing Statement [file jamanetwopen-e2517223-s001.pdf]

Data Sharing Statement

Liao. Disability Among School Children Across Districts of India. *JAMA Netw Open*. Published June 25, 2025. doi:10.1001/jamanetworkopen.2025.17223

Data
Data available: No

Additional Information
Explanation for why data not available: This study used administrative education data from the Unified District Information System for Education Plus (UDISE+) released by the Government of India's Department of School Education and Literacy (DoSEL) within the Ministry of Education
